# Evolution of resistance and disease tolerance mechanisms to oral bacterial infection in *Drosophila melanogaster*

**DOI:** 10.1098/rsob.240265

**Published:** 2025-03-12

**Authors:** Tânia F. Paulo, Priscilla A. Akyaw, Tiago Paixão, Élio Sucena

**Affiliations:** ^1^ Instituto Gulbenkian de Ciência, Oeiras, Portugal; ^2^ Faculdade de Ciências, Universidade de Lisboa, cE3c: Centre for Ecology, Evolution and Environmental Changes, Lisbon, Portugal; ^3^ Biologia Animal, Faculdade de Ciências da Universidade de Lisboa, Lisbon, Portugal; ^4^ CHANGE – Global Change and Sustainability Institute, Faculdade de Ciências da Universidade de Lisboa, Lisbon, Portugal

**Keywords:** innate immunity, resistance, disease tolerance, experimental evolution, *Drosophila*

## Abstract

Pathogens exert strong selection on hosts that evolve and deploy different defensive strategies, namely minimizing pathogen exposure (avoidance), directly promoting pathogen elimination (resistance) and/or managing the deleterious effects of illness (disease tolerance). However, how the host response partitions across these processes has not been directly tested in a single host–pathogen system, let alone in the context of known adaptive trajectories resulting from experimental evolution. Here, we compare a *Drosophila melanogaster* population adapted to oral infection with its natural pathogen *Pseudomonas entomophila* (BactOral), to its control population to find no evidence for behavioural changes but measurable differences in both resistance and disease tolerance. In BactOral, no differences were detected in bacterial intake or defecation, nor gut cell renewal. However, a measurable relative decrease in bacterial loads correlates with an increase in gut-specific anti-microbial peptide production, pointing to a strengthening in resistance. Additionally, we posit that disease tolerance also contributes to the response of BactOral through a tighter control of self- and pathogen-derived damage caused by bacteria exposure. This study reveals a genetically complex and mechanistically multi-layered response, possibly reflecting the structure of adaptation to infection in natural populations.

**Figure:**
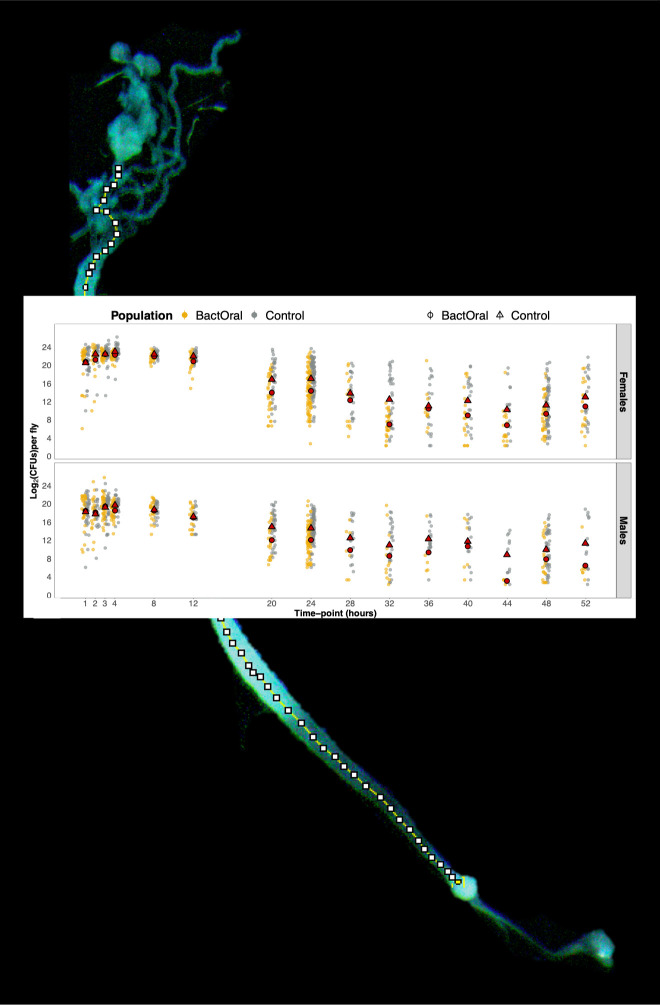

## 1. Introduction

Host–parasite interactions are major drivers of evolution [1,2]. In natural environments, hosts protect themselves from constant parasite threats through mechanisms that include behavioural strategies (e.g. avoidance) [3], the active reduction or elimination of pathogens (i.e. resistance) and/or the maintenance of homeostasis and fitness without interfering with pathogen burden (i.e. disease tolerance) [4–7]. While many studies in past decades have explored the mechanistic and genetic bases of these different host defence strategies in diverse organisms [8–10], few have focused on how these processes can be shaped by evolution [11–15]. Experimental evolution is a powerful method [16] that has enabled in multiple instances to address the impacts of different hosts on parasite fitness [17], of different pathogens on the host’s evolutionary trajectory [12,18,19], of immune priming [20,21] and of different routes of infection on host adaptation [19,22].

When feeding on pathogenic microorganisms [23,24], *Drosophila* deploys different responses that include avoidance behaviours [25,26], the barrier action of the peritrophic matrix [27], localized high acidity of the midgut [28], intensification of gut transit [29], local production of anti-microbial peptides (AMPs) [30] and reactive oxygen species (ROS) by gut epithelial cells [31,32] as well as increase of enterocyte delamination and renewal rates [33–36]. In addition, *Drosophila* can activate a systemic humoral response, when the pathogen manages to cross the gut epithelium into the haemolymph [37,38] or otherwise provokes a systemic reaction [39–41]. In the specific case of oral infection with its natural entomopathogen *Pseudomonas entomophila*, the production of toxins and virulence factors [42,43] causes translational arrest in host tissues and the inability to renew damaged gut epithelium [44,45].

In this work, we used a previously experimentally evolved outbred population of *D. melanogaster* (hereafter called ‘BactOral’) and investigated the mechanisms underlying its fast evolutionary response to a strong selection against oral *P. entomophila* infection [19,46]. We comprehensively tested multiple traits previously associated with immune response to oral infections. We quantified behavioural differences, pathogen loads and the immune response in BactOral and control populations under multiple conditions, to disentangle between the relative role of resistance (control of pathogen loads) and disease tolerance (host fitness independent of pathogen loads).

## 2. Results

### 2.1. Evolved population shows higher survival upon infection

**Figure 1.**
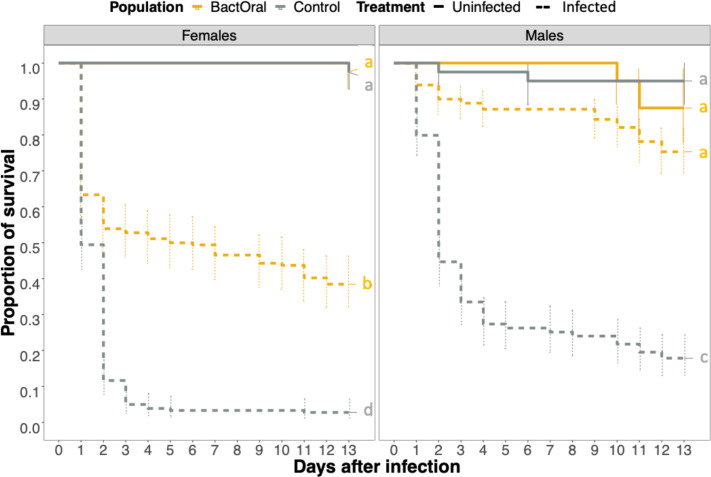
Survival upon infection with *P. entomophila*. Females (left) and males (right) of control (grey) and BactOral (yellow) populations were orally infected with a culture of *P. entomophila* OD_600_ = 50 (dashed lines) or a control solution (solid lines) for 24 h, after which they were flipped onto clean food and their survival measured daily. We identify a significantly higher survival after infection in BactOral (in both sexes) as compared with control, in a treatment-dependent manner. Results from multiple comparisons are shown as letters in the plots. Sample sizes were 40 uninfected and 180 infected individuals, for each sex per population.

In previous work [19,46], experimental evolution was conducted on an outbred population of *D. melanogaster* exposed to oral infection either with the bacterium *P. entomophila* or with a control solution (hereafter, control). Since then, these populations [46] have been kept under relaxed selection for over 80 generations. To understand to which extent this relaxation affected the response to the original selection regime, we measured the survival of control and BactOral populations upon *P. entomophila* oral infection. We found significantly higher survival of BactOral for both sexes (figure 1; infected control females versus infected BactOral females: *z* ratio = 9.123, *p* < 0.0001; infected control males versus infected BactOral males: *z* ratio = 9.909, *p* < 0.0001; electronic supplementary material, table S1). Moreover, these differences are not due to an inherent frailty of the control as compared with BactOral. Indeed, survival under uninfected conditions for both long-term longevity experiments (electronic supplementary material, figure S1 and table S2) and over the equivalent time period shows similar trends in both populations (figure 1; uninfected control females versus uninfected BactOral females: *z* ratio = 0.004, *p* = 1; uninfected control males versus uninfected BactOral males: *z* ratio = −1.080, *p* = 0.9611; electronic supplementary material, table S1).

Taking these results into account, we undertook a systematic description of multiple immune-related traits.

### 2.2. Evolved population has not changed bacterial uptake or defecation rates

**Figure 2.**
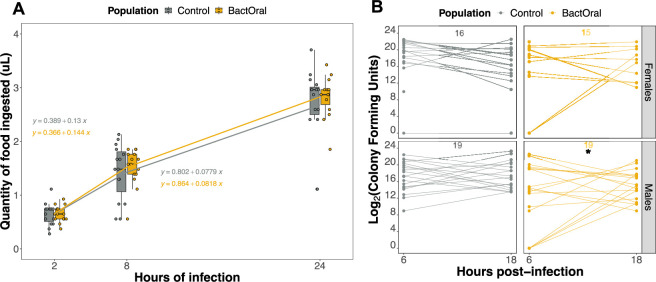
Evolved and control populations eat and defecate equivalent amounts of bacteria. (A) CAFE assay. Females of control and BactOral populations were fed individually on a *P. entomophila* suspension through a graduated glass capillary and the amount ingested (in µl) per fly was registered at 2, 8 and 24 h. A cumulative number of microlitres ingested per fly showed no statistically significant difference in amounts ingested by either population at the different time points. Linear regressions between averaged values eaten by each population between time points show a comparable decrease in feeding as the infection progresses (equations shown in plot for the periods 2−8 h (above lines) and 8−24 h (below lines)). From each population, 15 females were used. (B) Quantification of defecated bacteria. Females (top) and males (bottom) from control (grey) and BactOral (yellow) populations were fed *P. entomophila* for 3 h, after which defecated bacterial loads were estimated after 6 and 12 h. Lines connecting the two points provide a sense of individual rates of live bacteria defecation within this time window. Linear regressions between the two counts of the same fly show a significant decrease in the number of bacteria defecated between the two time points by males of BactOral (shown with an asterisk). Additionally, there is a significant difference in the number of BactOral flies that defecated below our detection threshold (zero CFU counts) at 6 h compared with 18 h and compared with control, for both time points. Sample sizes are shown at the top of each panel.

Our systematic approach started by screening behavioural traits that impact bacterial numbers in the gut: feeding and defecation. To test for differences in feeding behaviour, we performed a capillary feeding (CAFE) assay [47] on females of both control and BactOral populations at different time points over 24 h. We allowed individual flies to feed on a *P. entomophila* suspension mixed with food colouring in graduated glass capillaries and measured the amounts eaten by each female at 2, 8 and 24 h of exposure. We found no significant differences between the amount ingested by individuals of the two populations (power analysis for a *t*‐test: Cohen’s *d* = 1.06). Pairwise comparisons of their cumulative bacterial intake after 2, 8 and 24 hours exhibited a comparable quantity of total bacteria ingested (electronic supplementary material, table S3, and figure 2A; control versus BactOral at 2 h, *t* = 0.097, *p* = 1; at 8 h, *t* = 0.643, *p* = 0.9873; at 24 h, *t* = 0.574, *p* = 0.9925).

Additionally, we estimated the average slopes from linear regression where microlitres of bacteria eaten were modelled between different time points, concluding that the bacterial quantities ingested by both populations remain undistinguishable as the infection progresses. Thus, exposure affects the feeding ability of both populations to a comparable degree and notwithstanding the intrinsic individual variation common for both populations. Consequently, adaptation to oral infection with *P. entomophila* in the BactOral regime did not involve an alteration of feeding behaviour.

Considering the absence of significant differences in bacterial intake, an alternative would be that the BactOral population had evolved more effective bacterial defecation, which would reduce the time of pathogen permanence and proliferation in the gut. We established a protocol to measure live bacteria loads in the faeces, hence considering the number of bacteria defecated as a proxy for the defecation rate itself, assuming that no interaction between the two parameters exists. To this purpose, we took individual flies upon oral infection and quantified the colony-forming units (CFUs) defecated over a 6 h and the subsequent 12 h periods, post-exposure (figure 2B).

We observed a few instances where flies did not expel live bacteria during that period or did so in a quantity that fell below our detection threshold, resulting in a few counts of zero. Therefore, a zero-inflated model with a negative binomial distribution was fitted on the counts data (accounting for overdispersion) jointly with a binomial distribution on the zeros. For the counts portion of the model, none of the three factors significantly predicted the measured bacterial loads (electronic supplementary material, table S4; model parameters—population: *z* value = 1.324, *p* = 0.185; sex: *z* value = 0.073, *p* = 0.942; time point: *z* value = −0.359, *p* = 0.720). However, looking at the zero portion of the model, we find a significant effect of the population (population: *z* value = −2.407, *p* = 0.016; time point: *z* value = −0.006, *p* = 0.996).

We then performed *post hoc* pairwise comparisons on these regressions and identified a significant decrease in bacterial loads defecated by males of BactOral, between the two time points (males: *z* ratio = −2.719, *p* = 0.033), but no other significant effects were identified among bacterial counts between populations and/or time points, in a sex-dependent manner. However, when considering the zeros, we found a significant difference in the proportion of BactOral flies with undetectable amounts of bacteria in the faeces between the first and second time points (B6h–B18h: *z* ratio = 4.092, *p* = 0.0002), as well as compared with control at both time points (B6h–C6h: *z* ratio = 3.612, *p* = 0.002; B6h–C18h: *z* ratio = 3.530, *p* = 0.002; electronic supplementary material, table S4).

These results demonstrate that both control and BactOral populations defecate live bacteria in comparable amounts upon infection. Nonetheless, we detected that males of BactOral decrease the number of live bacteria they defecate as time progresses. This observation is corroborated by the difference in the number of ‘zeros’ found between both populations, with BactOral exhibiting more instances of expelling undetectable bacterial loads.

### 2.3. Evolved population shows a faster decrease and clearance of bacteria

Next, we studied different physiological responses to infection of control and BactOral, to estimate the roles of resistance and disease tolerance. First, we tested for structural changes in the gut itself that could influence the ‘carrying capacity’ of that structure and create difficulties in interpreting the quantification experiments. We measured the capacity of BactOral to regenerate its gut epithelium upon bacterial infection relative to control, using the initial length and extent of the shortening induced by infection [36], as a proxy. For this, we measured the total length of the guts of females, before and after 24 h of oral infection with *P. entomophila* and performed multiple pairwise comparisons on a linear model with mixed effects to pinpoint how population and infection status affected this trait. We found a shortening of approximately 2 mm in the total length of infected guts by comparing the post-infection time point of 24 h to the initial time point, prior to exposure, in both populations (electronic supplementary material, table S5 and figure S2; control before infection versus control 24 h post-infection: *t* = 6.274, *p* < 0.0001; BactOral before infection versus BactOral 24 h post-infection: *t* = 5.453, *p* < 0.0001). Nonetheless, no significant differences between populations in the total length of the guts could be detected both before and after infection (without infection: BactOral versus control, *p* = 0.6719; after 24 h of infection, *p* = 0.9925; power analysis for multiple linear regression: Cohen’s *d* for population = 0.71; *d* for treatment = 0.71; electronic supplementary material, figure S2).

**Figure 3.**
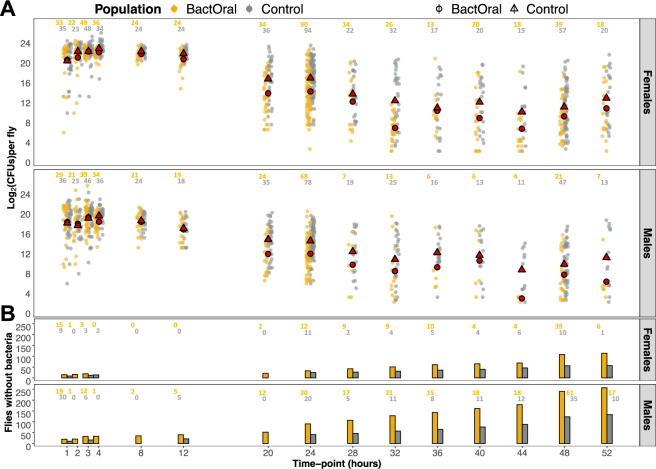
Bacterial load dynamics. Individual flies from control (grey) and BactOral (yellow) were collected upon exposure to *P. entomophila* at different time points for assessment of infectious loads of colony forming units (CFUs). After the end of the infection (24 h), flies were changed onto clean food and collection continued until 52 h post-infection. (A) Log_2_ of the number of CFUs found for females (top) and males (bottom) of either control (grey) or BactOral (yellow) populations, with the mean value at each time point evidenced in red (triangles for control and circles for BactOral). There are no detectable differences in bacterial loads between both populations until 20 h of infection, when BactOral starts to evidence a bigger decrease in bacterial counts. (B) A cumulative number of individual flies from which no CFUs were obtained increases as time progresses, with the BactOral reaching significantly higher final values. Numbers at the top of each panel correspond to the sample size at each time point, with the BactOral population in yellow and the control population in grey.

To explore the dynamics of bacterial proliferation inside the host, we performed a time-course characterization of bacterial loads during and after oral infection by counting the number of CFUs obtained from individual flies. In figure 3, we show the progression of bacterial loads from 1 h until 52 h post-infection (figure 3A) as well as the cumulative number of individuals without detectable live bacteria (figure 3B). The infection dynamics of both populations start with very high numbers of *P. entomophila* at early stages, confirming the absence of differences in the initial inoculum reported above (table 1; parameter *a*: 3.072 ± 0.016). Bacterial loads continuously decrease and, at approximately 20 h of infection, control and BactOral populations begin to differ significantly in a sex-dependent manner. From this point onwards, we observe a steady decrease in bacterial quantities in the two populations, always more severe in males than in females, as well as stronger in BactOral than in control flies (figure 3A). In addition, the number of flies that clear the infection also increases with time in both populations, but at a faster rate and reaching higher levels in BactOral (figure 3B). By 52 h post-infection, in both males and females, the number of ‘cleared’ flies in the BactOral population was twice as high as that of its control counterpart (figure 3B). Together, these analyses indicate that the evolved population eliminates bacteria faster and more efficiently than control.

**Table 1.** Parameter estimates for the model sigmoidal function fitted to the time course of bacterial counts. A sigmoidal function of four parameters (*a*, *k*, *h*, *b*) was fitted to the bacterial dynamics time course shown in figure 3A, for quantification of differences between control and BactOral CFU counts. Control females were used as reference on which to estimate the contribution of tested variables (variable ‘sex’: males; variable ‘population’: BactOral) on the parameters. Effects (sex and population) on parameters were considered significant when the 94% highest posterior density interval (hdi) did not include zero. The ‘ss’ parameter represents the estimate of the standard deviation of the normal error structure.

parameter	mean	s.d.	hdi 3%	hdi 97%
**ref. *k***	3.143	0.033	3.080	3.204
**ref. *h***	−2.309	0.144	−2.577	−2.043
**ref. *a***	3.072	0.016	3.043	3.104
**ref. *b***	2.361	0.034	2.298	2.425
**sex *k***	−0.053	0.068	−0.172	0.068
**pop. *k***	−0.108	0.046	−0.193	−0.021
**sex *h***	−0.330	0.227	−0.769	0.089
**pop. *h***	0.042	0.182	−0.301	0.376
**sex *a***	−0.130	0.038	−0.195	−0.061
**pop. *a***	−0.002	0.024	−0.047	0.043
**sex *b***	−0.147	0.066	−0.276	−0.035
**pop. *b***	−0.285	0.053	−0.384	−0.187
**ss**	3.671	0.062	3.552	3.785

In order to pinpoint more accurately the parameters contributing to this overall difference, we modelled the bacterial load and zero dynamics, and assessed differences in parameter estimates considering the initial load or inoculum (*a*), the load in the long-term or set-point bacterial load (*b*), the time point at which the load is reduced in half (*k*) and the highest rate of decrease or slope at time point *k* (*h*) (see §4 for details). In table 1, we show the mean estimates, standard deviation and high-density intervals (3% and 97%) for all four parameters of the fitted sigmoidal model for control and BactOral populations. Overall, these results show that sex has a significant effect on all curve parameters (*k* = −0.053 ± 0.068; *h* = −0.330 ± 0.227; *a* = −0.130 ± 0.038; *b* = −0.147 ± 0.066), reinforcing the notion of a strong sexual dimorphism in response to infections [48–50]. Additionally, populations differ on ‘time to half of the total’ (*k* = −0.108 ± 0.046) and the final load (*b* = −0.285 ± 0.053), but not on the initial load (*a* = −0.002 ± 0.024) nor on the highest rate of decrease (*h* = 0.042 ± 0.182).

Because this time-course experiment yielded a significant amount of zero counts, corresponding to individual flies for which no CFUs could be counted, we treated this as an independent dataset for which we developed a separate model focused on infection clearance (table 2). With that, we found that *a*_1_ (final fraction of zeros) was significantly affected by both sex and population (with no observable overlap between the mean estimates for each condition) (*a*_1_ females = −0.781 ± 0.07; *a*_1_ males = −0.061 ± 0.069; *a*_1_ control = −0.700 ± 0.070; *a*_1_ BactOral = −0.144 ± 0.069), with more BactOral individuals having cleared the infection by the end of the experiment, and males more so than females. There were significant overlaps between the estimates of the remaining parameters, evidencing a difference in clearance rate only in the late stages of bacterial infection. In sum, these data align with those of bacterial load dynamics, whereby flies from the BactOral population clear *P. entomophila* faster and better, from 20 h of exposure onwards.

**Table 2.** Parameter estimates for the model logistic function fitted to the zero counts of the time course of bacterial counts. An adapted logistic function of four parameters (*a*_0_, *a*_1_, *k*_0_, *k*_1_) was fitted to the progression of zero counts shown in figure 3B for the quantification of bacterial clearance differences between control and BactOral. Single estimates were generated for the contribution of each variable (sex: females and males; population: control and BactOral) to the model parameters. Effects were considered significant when the mean estimate ± s.d. did not overlap for the same parameter between each variable, which is the case for *a*_1_ when considering population and sex. High-density intervals of 3% and 97% show that all our estimated parameters have a high-density probability (i.e. credibility) as they are all included in them.

parameter	mean	s.d.	hdi 3%	hdi 97%
***a*_0_ control**	−1.214	0.570	−2.262	−0.127
***a*_0_ BactOral**	−0.731	0.570	−1.816	0.324
***a*_0_ female**	−0.984	0.569	−2.073	0.075
***a*_0_ male**	−0.959	0.569	−2.064	0.079
***a*_1_ female**	−0.781	0.071	−0.914	−0.645
***a*_1_ male**	−0.061	0.069	−0.188	0.074
***a*_1_ control**	−0.700	0.070	−0.833	−0.570
***a*_1_ BactOral**	−0.144	0.069	−0.268	−0.010
***k*_0_ female**	0.086	0.097	−0.096	0.269
***k*_0_ male**	0.085	0.095	−0.088	0.268
***k*_0_ control**	0.066	0.097	−0.114	0.250
***k*_0_ BactOral**	0.104	0.095	−0.076	0.281
***k*_1_ female**	−0.161	0.103	−0.354	0.030
***k*_1_ male**	−0.232	0.103	−0.426	−0.037
***k*_1_ control**	−0.161	0.101	−0.355	0.027
***k*_1_ BactOral**	−0.231	0.105	−0.426	−0.033
***t*_0_ female**	0.152	0.119	−0.071	0.372
***t*_0_ male**	0.253	0.116	0.031	0.462
***t*_0_ control**	0.168	0.119	−0.047	0.397
***t*_0_ BactOral**	0.238	0.116	0.020	0.453

These results evidence that bacterial loads are differentially controlled between BactOral and control populations, despite the equivalent initial bacterial inocula (figure 2A, table 1) and rates of live bacteria defecation (figure 2B). The differences in the number of detected live bacteria inside flies suggest that a direct bacterial control mechanism is operating more efficiently in BactOral individuals.

### 2.4. Differences in anti-microbial peptide expression are consistent with distinct bacterial load dynamics

**Figure 4.**
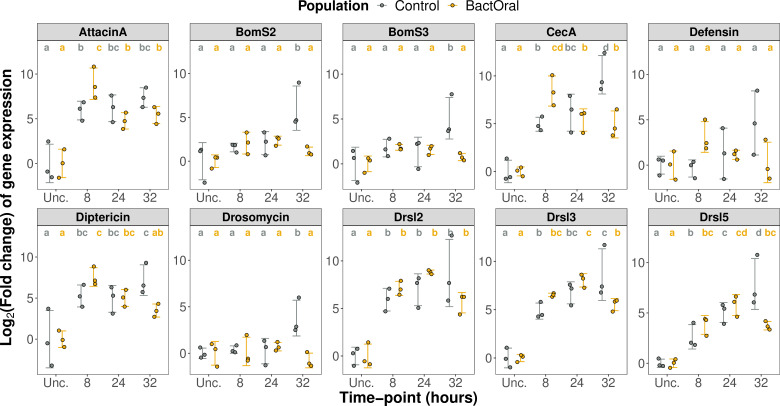
AMP expression differs between populations. Guts from control (grey) and BactOral (yellow) female flies (3 samples per population/time point, each containing RNA of 10 guts) were dissected before exposure (‘Unc.’) and at different time points after oral infection (8, 24 and 32 h) with *P. entomophila*. Relative gene expression, normalized to housekeeping gene *EIF2,* was performed for a representative panel of 10 AMPs. We detected a significantly higher upregulation in BactOral compared with control for *AttacinA* and *CecropinA* at 8 h of infection. Results from these comparisons are shown in the letters above the plots.

In an attempt to pinpoint which resistance mechanisms contribute to the differences in bacterial clearance rates observed between populations, we infected female flies from control and BactOral and dissected their guts at different time points. We collected samples before exposure (unchallenged) and after 8, 24 and 32 h of infection with *P. entomophila*, having flipped the flies onto clean food at the 24 h time point. We quantified gene expression levels for a panel of 10 AMPs covering different molecular families, dedicated pathways and pathogen specificities, including *Attacin-A*, *Bomanins Short 2* and *3*, *Cecropin-A1*, *Defensin*, *Diptericin-A*, *Drosomycin* and *Drosomycin*-like *2*, *3* and *5* (figure 4; electronic supplementary material, tables S6, S7, and figure S3).

We first verified if the baseline levels of AMPs (i.e. expressed under homeostatic conditions) differed between populations, by relativizing expression levels to the control population at the unchallenged time point. With this approach, we established that BactOral and control populations express comparable amounts of 8 AMPs when unchallenged, the two exceptions being *Cecropin-A1* (*CecA*) with lower expression levels (*t* = 2.892, *p* = 0.0445) and *Drosomycin-like 5* (*Drsl5*) with higher levels (*t* = −4.686, *p* = 0.0094) (electronic supplementary material, figure S2) in the control compared with the BactOral population.

In addition, and since our main goal was to characterize the temporal dynamics of AMP expression in both populations, we performed the analysis for the entirety of the time course using the unchallenged time point as reference (power analysis for a *t*‐test: Cohen’s *d* = 0.62; figure 4 and electronic supplementary material, tables S6 and S7). Overall, we detect consistent dynamics with the reduction of expression happening sooner (by 32 h) in BactOral than control across 7 of the 10 AMPs tested, the exceptions being *Attacin A*, *Defensin* and *Drosomycin-like 2* (*Drsl2*) (figure 4). This earlier downregulation in BactOral is relative to control flies (at 32 h) of *BomaninS2* (*BomS2*) (*t* = 4.347, *p* = 0.0047), *BomaninS3* (*BomS3*) (*t* = 4.035, *p* = 0.0089), *CecA* (*t* = 4.529, *p* = 0.0010), *Diptericin* (*t* = 2.613, *p* = 0.0439), *Drosomycin* (*t* = 4.795, *p* = 0.0056), *Drosomycin-like 3* (*Drsl3*) (*t* = 3.194, *p* = 0.0105) and *Drsl5* (*t* = 4.266, *p* = 0.0021). Furthermore, we detected a significantly higher upregulation of *AttacinA* and *CecA* in BactOral at 8 h of infection (*AttacinA*: control 8 h versus BactOral 8 h—*t* = −2.563, *p* = 0.0389; *CecA*: control 8 h versus BactOral 8 h—*t* = –3.034, *p* = 0.0123), consistent with the role of these peptides as main players against infections with Gram-negative bacteria [51,52]. A similar trend was observed for other tested AMPs such as *Defensin* and *Drsl2*, although these changes were not statistically significant (*Defensin*: control 8 h versus BactOral 8 h—*t* = −2.101, *p* = 0.231; *Drsl2*: control 8 h versus BactOral 8 h.p.i.*—t* = −0.899, *p* = 0.465).

Principal component analysis (PCA) supports these trends (electronic supplementary material, figure S4). The two main principal components (PC1 and PC2) evidence the time-dependent differences observed between populations by showing an initial indistinguishable grouping of both, followed by a dissimilar change in expression patterns at 8 h of infection that culminates with a maximum divergence between control and BactOral at the later time point (32 h).

### 2.5. Increase of anti-microbial peptide levels in the control population phenocopies BactOral response to infection

Having correlated the improved bacterial clearance of BactOral flies (figure 3) with significant differences in expression levels of AMPs between populations (figure 4), we sought to functionally associate these two observations. For that, we conducted a priming experiment to induce higher basal levels of AMPs by feeding flies a solution of heat-killed bacteria. These primed flies were, subsequently, infected orally with live *P. entomophila*, and followed daily for mortality. This exposure to dead bacteria has been shown to ‘artificially’ increase AMP levels [53–55]. We expect the priming with heat-killed bacteria to increase the basal expression of AMPs and thus provoke, particularly in control flies, an upregulation of the immune system, allowing test of the influence of this heightened condition in survival to oral infection.

**Figure 5.**
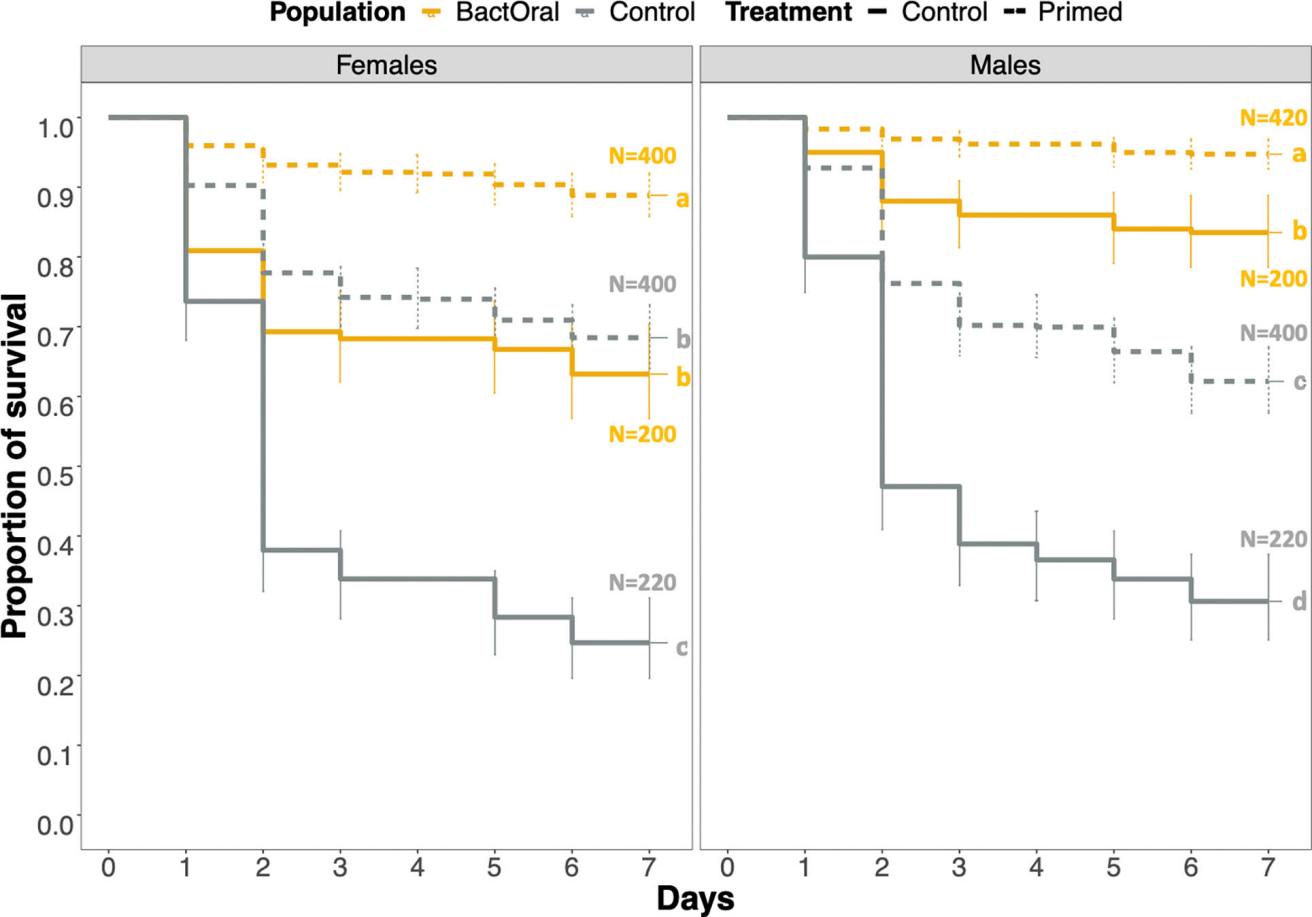
Control flies primed with heat-killed bacteria phenocopy BactOral survival to oral infection with *P. entomophila*. Control (grey) and BactOral (yellow) females (left) and males (right) were fed a solution of heat-killed bacteria for 2 h before oral infection (dashed lines) or directly infected with live *P. entomophila* (full lines) for 24 h, after which they were changed onto clean food and their survival measured daily. Previous exposure to heat-killed bacteria before infection increased survival in both populations, leading additionally control females to phenocopy BactOral survival levels upon infection. Results from multiple comparisons are shown as letters in the plots. Numbers at the end of each trajectory correspond to the sample size, with the BactOral population in yellow and the control population in grey.

Flies previously fed with heat-killed bacteria had increased survival upon infection with live *P. entomophila* in BactOral and control populations, confirming the stronger immune response expected upon priming (electronic supplementary material, table S8, and figure 5; BactOral females fed with live bacteria (control) versus BactOral females primed with heat-killed bacteria (primed): *z* ratio = 7.193, *p* < 0.0001; BactOral males treated with control versus BactOral males primed with heat-killed bacteria: *z* ratio = 4.368, *p* = 0.0001; control females fed with control versus control females: *z* ratio = 10.011, *p* < 0.0001; control males treated with control versus control males primed: *z* ratio = 7.299, *p* < 0.0001). Females from the control population primed with heat-killed bacteria increased their survival upon oral infection, virtually phenocopying BactOral infected with live bacteria, whereas in males, the same tendency is observed but to a lesser degree (electronic supplementary material, table S8, and figure 5; BactOral females fed with control versus control females primed: *z* ratio = 1.629, *p* = 0.3620 and BactOral males fed with control versus control males primed: *z* ratio = –4.547, *p* < 0001). This effect is supported by a time course of relative expression levels of IMD pathway key players, *Relish* (its devoted transcription factor) and its associated AMP, *Diptericin*, as well as the toll-activated AMP, *Drosomycin* (electronic supplementary material, figure S5). For both populations, we observed an increase in levels of *Diptericin* to a comparable degree upon exposure to heat-killed *P. entomophila*, but not of *Drosomycin*.

### 2.6. BactOral maintains higher survival upon immune response activation

**Figure 6.**
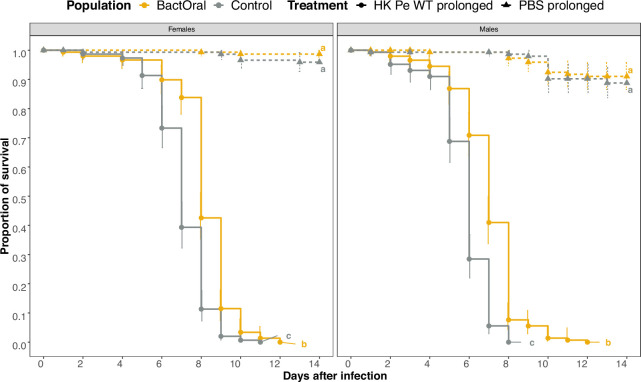
BactOral displays higher survival than control upon immune system activation without bacterial proliferation. Daily survival was measured for control (grey) and BactOral (yellow) populations fed a mixture of food with heat-killed bacteria or PBS continuously. BactOral females and males exhibit slower mortality compared with control flies until around day 8. This difference fades away for both sexes after that period. Survival comparisons between populations and treatments are shown as letters at the end of the curves. Sample sizes were 150 individuals for each sex, per population, per treatment.

To ascertain whether disease tolerance may also contribute to BactOral increased survival upon oral infection, we continuously fed flies with a mixture of heat-killed *P. entomophila* and followed survival (figure 6).

With this set-up we verified that both females and males of BactOral survive significantly more than control after the specific damages induced by constant exposure to heat-killed bacteria. Importantly, the survival differences detected between populations occur up until the time at which flies were selected to reproduce during the experimental evolution protocol (10 days upon infection), evidencing the adaptive nature of this response (electronic supplementary material, table S9, and figure 6; control-heat-killed versus BactOral-heat-killed females: *z* ratio = 6.579, *p* < 0.0001; males: *z* ratio = 9.612, *p* < 0.0001). No significant differences exist under control conditions (control-PBS versus BactOral-PBS females: *z* ratio = 1.392, *p* = 0.504; males: *z* ratio = 0.587, *p* = 0.936).

## 3. Discussion

In this work, we enquired systematically for the bases of adaptation of one line of *D. melanogaster* evolved against an oral bacterial infection, considering the three general layers that constitute the immune response: behaviour, resistance and disease tolerance [7,56]. Upon experimental evolution of an outbred population of *D. melanogaster* against oral infection with *P. entomophila*, and after approximately six generations, the selected population (BactOral) increased survival upon infection [19]. This phenotype was maintained throughout the selection experiment but, since then, the population has been kept for more than 80 generations under relaxed selection. Despite the fact that no physiological costs associated with this response could be detected in any of the original four replicates [46], we tested the immunocompetence of one selected representative replicate per evolved population. The average survival rate after oral infection of flies from BactOral was approximately 63% (50% for females and 75% for males) and approximately 10% in the control population (5% for females and 15% for males). Considering that at the end of selectionBactOral survival reached 90% [19], this partial decrease in overall survival after infection with bacteria can potentially underestimate the contributions of each of the different mechanisms behind adaptation. Importantly, these losses could be attributed to genetic drift acting on the populations during the long relaxed selection period they sustained. Notwithstanding, we established that it is methodologically possible to ascertain the relative importance of behavioural, resistance and disease tolerance mechanisms.

### 3.1. No role for behavioural or structural differences

It is well described that the route of infection is of paramount importance to the outcome of host–parasite interactions. In *D. melanogaster*, the mode of entry of a bacterial pathogen into the host’s body elicits different physiological responses [57–61], which can start by the avoidance of the pathogenic substrate, in the case of infection through the oral route [62,63], both by inhibiting feeding or choosing oviposition sites according to bacterial composition [53,62–64]. *P. entomophila* is a natural pathogen of *Drosophila* that causes high mortality when ingested, across diverse genetic backgrounds and conditions [23,43,44,65,66]. Thus, it is plausible to consider that fasting could constitute a general response to avoid or decrease the severity of an oral infection. The simplest behaviour that could explain the observed differences in survival between populations would be that BactOral flies evolved a reduced intake during exposure to bacteria, thereby reducing ‘inoculum’ size. Indeed, fasting is a prevalent response to infection and behavioural avoidance is a common strategy to limit exposure to infected substrates in different systems [3,62,63]. However, we showed that similar amounts of bacteria are ingested by females of both populations (figure 2A) [23,67]. Moreover, we substantiate the findings from the CAFE assay with the bacterial loads time course, which show equivalent amounts of *P. entomophila* colonies inside infected flies at early time points. Our results indicate that behavioural inhibition of bacterial intake was not one of the mechanisms that evolved in BactOral flies, eliminating fasting as a component of the adaptation to *P. entomophila* exposure.

Previous reports have shown that pathogen intake can affect gut function and structure [34–36,45] as well as influence the dynamics of exposure by altering defecation rates [29]. However, we could not find evidence for any differences in these features between control and evolved populations. Firstly, there are no changes in the gut length in steady-state conditions that would somehow enable the BactOral population to face *a priori* bacterial infection more favourably. Increased enteric cell renewal, which often leads to a temporary shortening of the gut length [36], has been described as one of the main mechanisms of *D. melanogaster* defence against oral bacterial infection [34,36,45]. In our experiments, we detected a significant decrease in the total gut length after 24 h of oral infection, but this change was equivalent in both populations, not supporting a role for altered renewal rate in BactOral adaptation.

These experiments, however, do not completely discard the hypothesis of differential gut structure underlying the adapted phenotype of BactOral. For example, proportions of gut cell types can change upon infection [68] and such a qualitative shift could contribute to higher fitness upon exposure to *P. entomophila* in the adapted population. Also, it could be that relevant changes in gut length occur at later time points. It has been previously shown that infection-induced gut cell loss starts as early as 2 h after exposure, with a return to 75% of the total original gut length after 24 h [36]. This time window is well within the boundaries defined in our set-up. Another important aspect to note is that renewal capacity is sexually dimorphic under diverse physiological contexts [69,70]. For instance, females have higher gut cell renewal rates than males upon oral infection with *P. carotovorum* 15 [71], a bacterium that provokes higher mortality in males than females, contrary to oral infection with *P. entomophila* (see Results). The disparity between those and our observations may be explained by the different mechanisms of virulence employed by these two pathogens to infect *D*. *melanogaster* [45]. Be that as it may, we envision further experiments testing for potential differences in gut delamination rates between evolved populations at distinct time points.

BactOral flies also did not expel pathogenic bacteria from their guts differently from control. However, males of BactOral show a decrease in the number of live bacteria defecated between 6 and 18 h post-exposure, to some extent evidencing improved bacterial clearance and suggesting a role for resistance. Since our protocol only detects live bacteria, we could potentially be missing an important component of the response and future work should quantify defecation of alive and dead bacteria, for example through fluorescence microscopy, to compare populations with regard to the contribution of overall peristalsis to the elimination of the total gut content [29]. In addition, considering the slightly higher number of BactOral flies that defecated live bacteria below our detection limit (zeros), we hypothesize that this population either retains *P. entomophila* for longer inside guts before beginning to expel it (pointing to higher disease tolerance) or reduces bacterial loads more effectively at earlier stages, even though, with time, bacterial proliferation recovers (again suggesting stronger resistance).

### 3.2. A role for resistance

To further test the suggested role for resistance, we characterized the bacterial load dynamics in evolved populations over 52 h and determined that BactOral flies eliminate pathogens in a more efficient way than control. This significant change in bacterial loads indicates that BactOral has evolved higher resistance by an earlier control and better clearance of gut bacterial loads. As the chosen statistical model to disentangle these differences was applied to the logarithm of mean bacterial counts at each time point, it is possible that information regarding intra-populational variation is diluted in the analysis. Also, the recently uncovered bactericidal effects of a core fly food component (i.e. methylparaben [72]) might be skewing our initial bacterial load counts towards a higher threshold, although we estimate that effect to equally influence both populations. However, our conclusions from the comparison of the bacterial counts are further strengthened by the separate analysis of the number of ‘zeros’ as a function of time, which is a proxy for bacterial clearance, supporting the observations concerning evolved differences in resistance.

Even though there is no discernible difference in the number of CFUs infecting both males and females between both populations until approximately 20 h of infection, by this time individuals from BactOral begin to exhibit gradually decreasing levels of bacteria. This trend aligns with the higher clearance rate observed in the adapted population (and higher in males than females) as measured by the increase in the number of flies from which we cannot grow colonies of *P. entomophila* (zeros). Flies from the control population control their bacterial loads (at around 20 h of infection) but do it at a slower pace and in a less effective way (i.e. many individuals exhibited high bacterial counts by the end of the experiment, at 52 h). Nonetheless, it is possible that our proxy for clearance (i.e. flies classified as ‘zeros’) could be overestimated due to a low detection threshold. Indeed, it has been shown that *D. melanogaster* can endure chronic bacterial infections [73–75], albeit at rather low levels, which can be the case in our experimental set-up. However, this holds true for both populations, hence not invalidating our interpretation of a different bacterial clearance rate between genotypes.

Having shown that BactOral clears *P. entomophila* infection more rapidly, we hypothesized that this response could rely on an increased production of immune effectors, such as AMPs. Analysis of AMP gene expression in guts showed that in the unchallenged condition, only two AMPs (*CecA1* and *Drsl5*) differed in expression between populations (downregulation and upregulation, respectively, in BactOral), indicating that the base levels of AMP expression are unlikely to have been a major target of selection. The detected expression difference of *Drsl5*, a gut-specific AMP peptide, could represent a pre-emptive resistance mechanism acquired by BactOral, by establishing a more antagonistic environment for *P. entomophila* proliferation in the guts of these flies after ingestion.

At 8 h of exposure, two AMPs show a significantly faster upregulation in BactOral, while, later on (32 h), seven of the panel members display a pattern of faster downregulation. These results suggest that a two-step process might be taking place, whereby the response is initially (8 h) more aggressive and is turned off sooner (32 h). This pattern of response is consistent with both resistance and tolerance mechanisms. On the one hand, the early acute response permits a more effective pathogen elimination (resistance). On the other hand, a faster shut down of the response, here measured as AMP production but potentially extendable to other mechanisms (i.e. broad sense inflammation), should limit the secondary effects of prolonged immune responses (immunopathology). Interestingly, *Drosomycin-like* genes (*Drsl2*, *Drsl3* and *Drsl5*), whose expression is specific to the gut epithelium and is regulated by JAK-STAT [35], show upregulation after 8 h of infection in both populations. For all three genes, there is a slight tendency for a faster response that could represent a more effective bacterial control in the BactOral population and a faster downregulation at the 32 h time point that could limit self-inflicted damage. Likewise, we observed a similar process of downregulation of other AMPs specifically deployed to fight off infections with Gram-negative pathogens (e.g. *CecA1* and *Diptericin*) at the 32 h time point. In contrast, some AMPs typically associated with response to Gram-positive bacteria (e.g. *BomS2*, *BomS3* and *Drosomycin* [51,76,77]) do not exhibit significant upregulation after exposure to *P. entomophila* until 32 h post-infection and only in the control population.

Strikingly, and even though *Diptericin* has been shown to be deployed by *D. melanogaster* to resist *P. entomophila* infection [67], we did not detect a difference in the relative expression of this gene between control and BactOral until after 24 h of infection. At this time point, adapted flies are already returning to basal levels of AMP production whereas control flies keep actively fighting off the infection, *sensu* AMP production.

The same pattern is observed in the priming experiment (figure 5 and electronic supplementary material, figure S5). After priming with heat-killed bacteria and subsequent exposure to live bacteria, both populations maintain an equivalent gene expression increase until 8 h but, from 24 h onwards, BactOral shows again an earlier downregulation of *Diptericin* and a quicker return to homeostatic levels of expression (electronic supplementary material, figure S5). In brief, the dynamics of *Diptericin* expression mimics the expected effect of priming by establishing higher basal levels of this AMP with which to counteract an incoming infection. This is not seen for the AMP *Drosomycin*, which again is consistent with our previous result that this AMP is not deployed against infection with *P. entomophila* (figure 4 and electronic supplementary material, figure S3).

With this, we reinforce the notion that the process of earlier AMP downregulation could be an active response rather than a consequence of lower bacterial loads. This points to a potential role for disease tolerance mechanisms in this population through the prevention of AMP-induced immunopathology.

### 3.3. A role for disease tolerance

Maintenance of fitness in the face of stress-inducing or homeostatic conditions is ensured by tissue damage control mechanisms [5]. Furthermore, these damage control responses can be activated in the event of an infection, either due to the direct action of pathogens (i.e. injury-inducing toxins) or indirectly from immune-derived damage (i.e. immunopathology).

A putative role for disease tolerance was tested by measuring the survival of the evolved populations to prolonged exposure to heat-killed bacteria following an established protocol designed to fix the effects of resistance and best explore the phenomenon and mechanisms of disease tolerance [78]. This protocol provided a means by which to test the response to damage (tolerance), whether provoked by the pathogenicity of the bacteria or by the host’s own response, independently of active pathogen elimination (resistance). Although we hypothesize that the heat-killing treatment was insufficient to denature all the virulence factors present in the suspension, we are yet to disentangle the relative contribution of virulence factors versus immunopathology to the longevity measurements. A similar longevity was observed for females and males of control and BactOral after feeding on heat-killed bacteria, but an interesting trend appeared when comparing survival profiles (figure 6). We found that BactOral survived better to the stress of prolonged exposure to heat-killed *P. entomophila*, specifically under an evolutionarily relevant timeframe, that is, the period when flies reproduced during the selection experiment. Physiological responses measured approximately at 10 days post-infection (the age at which populations were selected to reproduce) can potentially represent evolutionary costs [79–82], which are not the focus of this study. Although both control and BactOral individuals fully succumbed to the chronic exposure to heat-killed bacteria, significant differences in survival distinguish the two populations.

Additionally, together with the gene expression data presented in figure 4, showing that BactOral represses the activation of immune effectors earlier upon infection, it is tempting to hypothesize that a similar process happened during the course of this experiment, and a role may be attributed to tighter control and negative regulation of immune over-activation.

The improved response of adapted flies to exposure to heat-killed bacterial substrate can be explained by a higher capacity to withstand damage, whether (i) provoked by their own immune response or (ii) caused by virulence factors present in inactivated bacterial supernatant. Additionally, process (i) could be correlated with the previously identified tighter immune repression. Disentangling the relative contribution of these mechanisms to the overall adapted phenotype would need further empirical characterization.

In sum, we identified resistance and disease tolerance mechanisms as putative targets of experimental evolution for increased survival from *P. entomophila* oral infection. Our observations align with previous work that identifies a positive genetic correlation between these two distinct immune defence strategies [83]. The increased survival of an experimentally adapted population to oral infection directly relates to its capacity to decrease bacterial loads after infection, independently of bacterial feeding rate, gut renewal capacity and bacterial defecation. Previous work found resistance to be targeted by selection upon immune challenge in *D. melanogaster* [80,84]. Our work adds to this knowledge by approaching the other mechanisms at play in immunity, namely disease tolerance and behaviour, in the framework of a single evolutionarily coherent system. We report one additional case of resistance evolution that exhaustively dissects the different processes by which immunity may evolve in populations fighting off persistent infections. In addition to resistance, we find a quantifiable role for disease tolerance in the evolved response of BactOral, revealing the complex nature of this adaptative process [83], which is likely to rely also on improved tissue repair coupled with a more controlled deployment of damage-inducing immune effectors, such as AMPs [85,86]. Future work will focus on identifying the specific mechanisms of disease tolerance, their physiological mode of action and interactions with resistance and their potential evolutionary repeatability.

## 4. Methods

### 4.1. Maintenance of *Drosophila* populations under relaxed selection

Experimentally evolved control and BactOral populations were initially derived from an outbred population of *D. melanogaster*, described in previous studies [19,46]. During experimental evolution, four replicates of each selection regime were maintained by orally infecting 310 females and 310 males per replicate, with a suspension of *P. entomophila* previously determined to cause approximately 66% of mortality in the starting population. After selection was terminated, populations were kept at high census (1500−2000 individuals per cage), allowing for maintenance of high genetic variability and optimal larval development. For all generations, flies were kept under constant temperature (25°C), humidity (55−65%) and light–darkness cycle (12 : 12), on a standard cornmeal–agar medium, consisting of 4.5% molasses, 7.5% sugar, 7% corn flour, 2% granulated yeast extract, 1% agar and 0.25% nipagin, mixed in distilled water. Control and BactOral were under selection for 24 generations and under relaxed selection for approximately 80 generations, after which one replicate of each regime was singled out to perform the experiments described.

Each generation cycle lasted approximately three weeks, during which flies eclosed and remained undisturbed until they were 7−8 days old. At this time, fresh food was placed in the cages for 2 days, which allowed for oviposition of retained eggs. After this period (when flies were 9−11 days old), similarly to the protocol applied for selection, new fresh food was placed in the cages for controlled egg-lays (1–3 h long) to establish the next generation.

### 4.2. Pathogen culture and infections

Oral infection protocol with *P. entomophila* (rifampicin-resistant strain kindly provided by Bruno Lemaitre) was adapted [19]. Briefly, single bacterial colonies were grown in kick-start cultures (5 ml of Luria-Bertani broth (LB)) for approximately 8 h after which they were transferred into larger volumes (1 l of LB) for overnight growth, both periods at 29°C. After centrifugation and resuspension of bacterial pellets, concentration was adjusted to OD_600_ = 100 and finally diluted 1 : 1 with a 5% sucrose solution. For infection, 3- to 5-day-old flies were separated by sex into groups of 20 with the use of CO_2_ (at least 24 h before the start of the experiments) and allowed to feed on a filter disc embedded with the bacterial solution described above for 24 h (for survival assays) or for the amount of time specific to each experiment (see Results). After this period, infected flies were flipped onto clean food and survival was scored daily. Controls were fed a 1 : 1 LB/phosphate-buffered saline (PBS) : 5% sucrose solution.

For the priming experiment, a solution of *P. entomophila* at OD_600_ = 100 was previously heat-killed for 1 h at 55°C, mixed 1:1 with a 5% sucrose solution and fed to flies for 2 h. The heat-killed culture was plated to confirm the absence of bacterial growth. Flies were transferred into tubes containing a live solution of *P. entomophila* for 24 h, after which they were changed back to clean food and their survival scored.

For the experiment with prolonged exposure to heat-killed *P. entomophila* bacterial preparation and heat-killing protocol were performed as described above, but the final dead bacteria suspension was mixed 1 : 1 with standard fly food and dispensed into fly vials. To the final solution, extra agar powder was added to account for viscosity and to optimize texture and left to cool down and solidify for at least 2 h, before being fed to flies. The same protocol was performed for the control treatment, in which fly food was mixed with sterile PBS. Exposure to these treatments was constant throughout the experiment, during which replicates of 15 females and 15 males were co-housed in vials, exclusively feeding on the dead bacteria or PBS diets. Survival scoring began at the start of exposure and lasted 14 days. During the treatment, flies were frequently flipped onto fresh food mixture and survival scoring continued daily. The heat-killing protocol was tested as above.

Survival analysis was done for the multiple experiments by fitting data with Cox proportional hazards models (with *coxme* or *coxph* functions from the *survival* package [87]) using population, treatment and sex as fixed factors and replicate (or replicate nested within a block) as a random factor. To assess the effect sizes of each factor, type 3 ANOVAs (ANOVA from the *car* package [88]) were run on the survival models. Additionally, multiple comparisons with *Tukey* adjustments were done on all the models to disentangle which specific pairwise comparisons were significantly different (*emmeans* function [89]). Finally, the *cld* function of the *multcomp* package [90] (with *Sidak* confidence-level adjustments) was used to summarize the results of the multiple comparisons across conditions. Survival models used were:

— immunocompetence (figure 1): *coxme*(~Population × Treatment × Sex + (1|Replicate));
— priming (figure 5): *coxme*(~Population × Treatment × Sex+(1|block/Replicate)); and
— immune over-activation (figure 6): *coxme*(~Population × Treatment × Sex +(1|Replicate), data = survival_HKexposure).

### 4.3. Bacterial defecation assay

To have a measure of the rate of gut purge/defecation a change was introduced to the bacterial infection protocol, whereby the feeding period was reduced to a 3 h period. After this time, flies were surface sterilized and separated individually into plastic spectrophotometry cuvettes, previously filled with fly food. After 6 h, the same flies were changed into fresh cuvettes where they remained for an additional 12 h, after which they were discarded. Bacterial quantification was performed for both time points by washing the interior surface of the cuvettes with PBS and plating, as described below.

Statistical analysis on the number of defecated bacteria was done by using a zero-inflated model (zeroinfl function [91] from the pscl package [92]), which allowed fitting of a negative-binomial distribution on the counts data and of a binomial distribution on the zeros (zeroinfl (formula = Counts ~ Population × Sex × Timepoint | Population × Timepoint, dist= ‘negbin’)). This approach was chosen because the dataset showed strong overdispersion and a significant abundance of ‘zeros’ (approx. 10%), which can be explained by different biological mechanisms (e.g. resistance and behaviour). Zero-inflated models enable the measurement of the contribution of different variables to both data partitions independently, namely counts and zeros.

To assess differences between specific pairwise comparisons we used *emmeans* with *Tukey* adjustments, on both distributions of the zero-inflated model. In the instances where flies died during either time point, their count at that time was removed from further analysis and the linear regression was not included in figure 2B.

### 4.4. Quantification of food intake by capillary feeder assay

To measure the amount of bacterial solution ingested by single flies, an adapted CAFE [47] assay was performed. We substituted the food of small fly vials with agarose, to ensure humidity, and placed a glass capillary in each lid, filled with 5% sucrose solution. We allowed individual flies to habituate to this experimental set-up for 24 h before the infection. After this period, capillaries filled with *P. entomophila* suspension (prepared as described before) were given to individual females to feed on, and consumption was estimated at different time points. To facilitate scoring in capillaries, the bacterial solution was mixed with blue food colouring.

Modelling of cumulative amounts of bacteria eaten over time was done with a generalized linear model with a gamma distribution (*glm*(Quantity~Population × Timepoint)) using the *glm* function from the base *stat* R package [93]. For better visualization of feeding over time, linear regressions were drawn between mean values of microlitres eaten cumulatively by each population at each time point, using the *stat_poly_eq* function of the *ggpmisc* package [94]. Finally, multiple comparisons were done with *emmeans* [89], using *Tukey* adjustments, to disentangle differences between populations across time points.

### 4.5. Measurement of gut length

Females were individually dissected in PBS, and their guts removed for imaging by adapting an established protocol [95]. Dissections started by fixing flies in the head with a Minutien pin and gradually separating the gut from the remaining tissues, in a posterior-to-anterior direction. After the gut was exposed, we added a few drops of a solution of 5% acetic acid and 50% EtOH, which slightly whitens the tissues, allowing for better visualization and a slight tissue fixation. After having isolated the guts, they were individually transferred to a coverslip and photographed using a uEye camera installed on an Olympus SZX7 stereoscope.

Images were processed in FIJI v. 2.0.0 and measurements were collected by drawing consecutive transects along the total length of guts, as illustrated in electronic supplementary material, figure S2B. Each individual gut measurement was repeated three times to account for errors in the manual drawing of the transects and averaged to obtain a final gut length in millimetres (mm).

Differences in lengths of guts between populations were analysed with a linear mixed-effects model (*lmer*(Length~Population × Treatment + (1|Block)) with *lmer* from the *lmerTest* package [96]. The model was followed by multiple comparisons with *emmeans*, with a *Tukey* adjustment, for assessment of individual pairwise differences.

### 4.6. Quantification of bacterial loads

To quantify bacterial loads, we adapted an established protocol [19,97]. In short, individual flies were surface sterilized (washed in EtOH 70%, bleach 60% and two washes with MilliQ water) and placed in 96-deep-well plates, where they were homogenized with glass beads in 50 µl of LB using a TissueLyser II (Qiagen). Individual samples were serially diluted five times with 1 : 10 dilution steps, plated as 4 µl droplets in LB + rifampicin, and left to grow overnight at 29°C to optimize colony size for counting. For the bacterial defecation assay, cuvettes that contained flies for each of the time points were filled with 1 ml of PBS and incubated at room temperature for 15 min, after which each sample was diluted up to four times (1 : 5 dilutions) and plated as droplets in LB + rifampicin.

Analysis of the time course of infection progression was performed by dividing the data obtained from the bacterial quantification into ‘counts’ and ‘zeros’ and using different models to examine their dynamics in the evolved populations. For the counts, the modelling of the mean of the load dynamics during the time course was done using a sigmoidal function that considers four parameters: *a*, the log(initial load); *k*, the time point at which the load is half of the maximum value (in this case, *a*); *h*, the slope at the *k* time point (steepest slope of the curve); and *b*, the log final load). A linear model was applied to each of the parameters, which was fitted using a Monte Carlo Markov chain approach. To estimate the impact of sex and population on the likely distribution of the parameters, a normal error structure was assumed, using control females as a reference. These parameters were chosen as they represent different fundamental aspects of the host immune response, namely initial (*a*) and final (*b*) inocula, as well as the highest rate *h* (and time point, *k*) of bacterial elimination. For the ‘zeros’, we ran a modified logistic model with four parameters: *a*_0_, initial fraction of zeros; *k*_0_, rate of zero disappearance; *k*_1_, rate of zero re-appearance; *t*_0_, time point at which zeros re-appear; and *a*_1_, final fraction of zeros. Similar to the ‘counts’ analysis, linear models were used to assess the impact of sex and population on all four parameters.

### 4.7. RNA extractions and quantitative PCR

For RNA extractions, flies from control and BactOral populations were infected as described above and, at different time points, pools of 10 females were collected and homogenized in 500 µl Trizol. RNA extractions were performed using a Direct-zol™ RNA MiniPrep with Zymo-Spin™ IIC columns. After precipitation, a DNase I (RQ1 RNASE-FREE DNASE 1* from Promega^®^) treatment was done to all samples, followed by reverse transcription using a Thermo Scientific^®^ RevertAid H Minus cDNA kit. cDNA was finally diluted 1 : 5 for qPCR.

For quantification of gene expression, qPCRs were performed using SYBRTM Green Master Mix (Thermo Scientific^®^) and reactions ran on 384-well plates (Applied Biosystems^®^). The PCR conditions used in all experiments were initial denaturation/enzyme activation, 95°C for 10 min; followed by 45 cycles of denaturation, 95°C for 10 s; annealing, 60°C for 10 s; extension, 72°C for 30 s. Sequences of primers used for qPCRs are shown in electronic supplementary material, table S1. Either EIF2 (eIF-2alpha) or Rpl32 were used as a reference.

Analysis of gene expression differences was done with the relative quantification technique (DDCt) [98]. Briefly, from the average of technical replicates for each candidate gene we subtracted the average of the Ct values of the respective sample’s house-keeping gene (EIF2 or Rpl32) (DCt) and normalized this value to the DCt of the respective reference condition. For the time-course analysis, the reference was the ‘unchallenged’ time point while for comparisons between unchallenged conditions among populations, control was used as a reference. Finally, we used reverse logarithm to transform the final gene expression values into fold change levels (2^−DDCt^) and analysed the log_2_(foldchange) differences across Populations and/or Time points.

Statistical analysis on gene expression was done by running linear models (from base R stats [93]) on the log_2_(foldchange) values of each individual gene (*lm*(logfold_AMP ~ Population × Time point)), followed by an ANOVA (type three) from the car package [88]. To individualize significant pairwise comparisons, *emmeans* was run on the linear models (with a Benjamini–Hochberg adjustment). Finally, the *cld* function of the multcomp package [90] (with Sidak confidence-level adjustments) was used to summarize the results of the multiple comparisons across different conditions.

### 4.8. Statistical analysis and graphical representations

All datasets (excluding survival data) were tested for normality before further analysis, using the Shapiro–Wilk test (*shapiro.test* function of the *stat* R package), for determination of the most suitable type of statistical test to perform in each case. All model formulas used for statistical analysis are discriminated throughout §2. All graphical representations were performed using the *ggplot2* package [99].

Statistical analysis and graphics for all experiments, excluding analysis of the bacterial loads time-course modelling analysis (figure 5), were done on R v. 4.2.1, through RStudio [100] v. 1.3.959. For the bacterial dynamics time course, models were implemented and fitted in Python [101], using the probabilistic programming language package *pymc3* [100] v. 3.11.

Sensitivity (power) calculations for the CAFE and qPCR datasets were calculated using G*Power 3.1 [102].

Sensitivity analysis for the gut length dataset was performed by simulation, using Python packages *scipy* [103], *numpy* [104] and *statsmodels* [105]. Briefly, a synthetic dataset of the same sample sizes and structure as the real data but with a specified effect in both factors (treatment and genotype) was generated. Multiple linear regression was then performed and the significance (*p*‐value) of both factors was recorded. This was performed 10^5^ times, and the fraction of significant tests was recorded (power, independently for both factors). A numerical search for the minimum effect sizes that achieve 80% power on both factors was then performed.

## Data Availability

All relevant data are within the paper and the electronic supplementary material files [106].
